# Rhabdomyolysis and Cardiomyopathy in a 20-Year-Old Patient with CPT II Deficiency

**DOI:** 10.1155/2014/496410

**Published:** 2014-01-20

**Authors:** M. Vavlukis, A. Eftimov, P. Zafirovska, E. Caparovska, B. Pocesta, S. Kedev, A. J. Dimovski

**Affiliations:** ^1^University Clinic of Cardiology, Medical Faculty, Ss. Cyril and Methodius University, Mother Teresa 17, 1000 Skopje, Macedonia; ^2^Faculty of Pharmacy, Ss. Cyril and Methodius University, Mother Teresa 47, 1000 Skopje, Macedonia

## Abstract

*Aim*. To raise the awareness of adult-onset carnitite palmitoyltransferase II deficiency (CPT II) by describing clinical, biochemical, and genetic features of the disease occurring in early adulthood. *Method*. Review of the case characteristics and literature review. *Results*. We report on a 20-year-old man presenting with dyspnea, fatigue, fever, and myoglobinuria. This was the second episode with such symptoms (the previous one being three years earlier). The symptoms occurred after intense physical work, followed by a viral infection resulting in fever treated with NSAIDs. Massive rhabdomyolysis was diagnosed, resulting in acute renal failure necessitating plasmapheresis and hemodialysis, acute hepatic lesion, and respiratory insufficiency. Additionally, our patient had cardiomyopathy with volume overload. After a detailed workup, CPT II deficiency was suspected. We did a sequencing analysis for exons 1, 3, and 4 of the CPT II gene and found that the patient was homozygote for Ser 113 Leu mutation in exon 3 of the CPT II gene. The patient recovery was complete except for the cardiomiopathy with mildly impaired systolic function. *Conclusion*. Whenever a patient suffers recurrent episodes of myalgia, followed by myoglobinuria due to rhabdomyolysis, we should always consider the possibility of this rare condition. The definitive diagnose of this condition is achieved by genetic testing.

## 1. Introduction

Carnitine palmitoyltransferase (CPT) deficiencies are genetic disorders of mitochondrial fatty acid oxidation. Long-chain fatty acids are required for fueling the skeletal muscles and are only able to cross mitochondrial membrane after esterification with carnitine in a reaction with the enzyme CPT I. Inside the mitochondria, the fatty acid is reactivated to acyl-CoA from acylcarnitine and CoA with the help of CPT II in order to enter the *β*-oxidation cycle [1–3].

The CPT II deficiency presents in three different forms: lethal neonatal form; severe infantile hepatocardiomuscular form, and adult-myopathic form [1–8].

The adult-myopathic form is the most prevalent type of the disease with about 300 cases reported. The first description was made in 1973 by the brothers Di Mauro. The first symptoms most often occur between 6 and 20 years of age but the age of onset may be over 50 years and as early as 8 months of life [9, 10]. The symptomatology usually consists of recurrent attacks of rhabdomyolysis presenting as myalgias, muscle stiffness and weakness, and myoglobinuria. In rare situations, rhabdomyolysis may result in life-threatening complications such as acute renal failure and respiratory insufficiency secondary to respiratory muscles involvement [11, 12].

We are describing such a clinical case.

## 2. Case Presentation

We report a case of a 20-year-old man transferred to our clinic under suspicion of myocarditis. He presented with dyspnea, fatigue, myalgia, fever, and myoglobinuria. The patient reported those three days before becoming symptomatic, he was subjected to a more intense physical work (unloading a truck). The next day he felt like coming down with a cold, he felt weak and had muscle pains. Later that day, he was admitted to the ER with fever (40°C) where he received a parenteral therapy with NSAIDs. After the parenteral therapy, he complained of worsening of the symptoms with strong myalgia, dyspnea, fatigue, sweating, and myoglobinuria.

Influenza B was identified, and increased markers of inflammation-fibrinogen, CRP, leukocytosis, plasma levels of CPK up to 114000 IU, CK-MB 5400 IU, and myoglobin up to 33000 IU were detected, resulting in acute renal failure (necessitating plasmapheresis and hemodialysis treatments), respiratory insufficiency (necessitating a noninvasive ventilation support), and a mild to moderate hepatic lesion.

During the whole period, the blood glucose levels were in the range of 3, 4-5, 4 mmol/L (lower normal limit), and no significant hypoglycemia was registered.

Muscle biopsy identified exacerbated chronic vasculitis with atrophy of certain muscle cells. The immunologic markers and hemoculture were negative. The pneumoslide was only positive to IgM for Influenza B.

**Figure 1:**
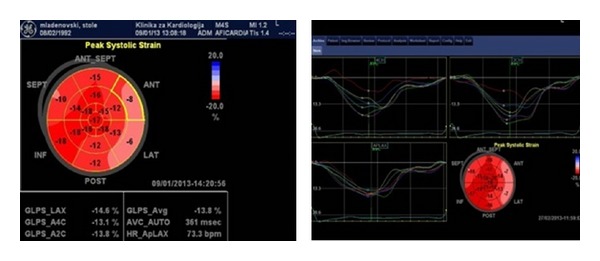
Strain analysis of the left ventricle.

The echocardiographic examination revealed enlarged left ventricle (LVEDd 65; LVEDs 48) with EF of 47% by the Simpson method. Strain analysis using speckle tracking identified GLPS average of −13.8% (Figure 1).

After one-month hospitalization period, the patient was stabilized and discharged from the hospital. During the nine-month follow-up period he was stable, completely recovered, and asymptomatic, except for a mildly impaired left ventricular systolic function.

His prior history revealed that the patient used to feel cramps and pain in the legs after prolonged walking in the childhood. He experienced a similar episode three years earlier after prolonged exercise (body building for several hours), exposure to a cold temperature, and infection (febrile illness), when he was hospitalized in another institution with severe signs of rhabdomyolysis (creatinine phosphokinase up to 42000 IU) and hepatic lesion (AST, ALT up to ×10 over the upper limit) and was diagnosed with cardiomyopathy with increased LV dimensions LVEDd > 60 mm, EF 45%. There were no signs of renal impairment at that time. He was stabilized and discharged from hospital. During attack-free periods, the patient was asymptomatic.

The patient had a deceased father from heart failure of unknown origin and a sister that died at the age of 18 months in unclear circumstances after “being sick all the time” when she received an injection for fever.

**Figure 2:**
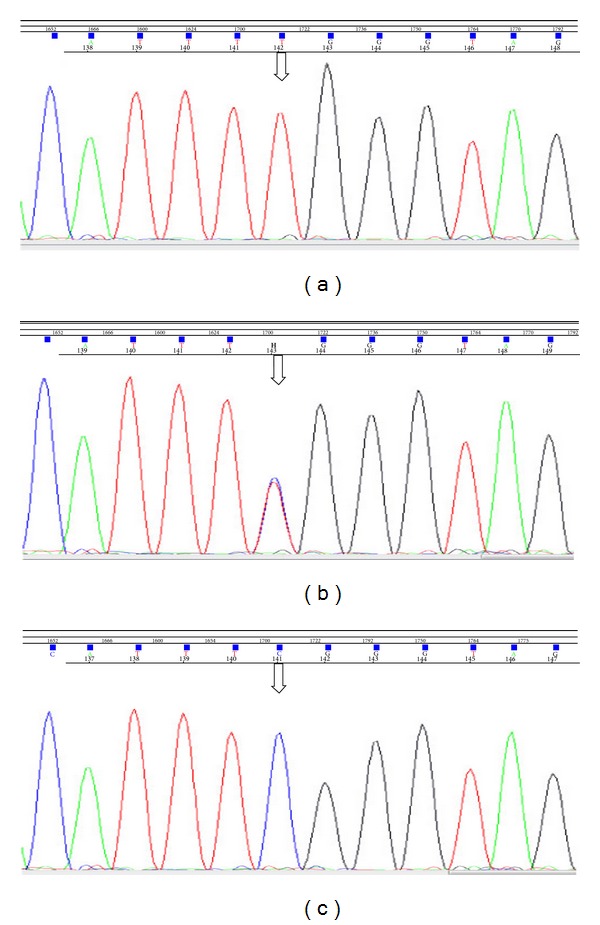
Sequencing analysis demonstrating the detection of TCG > TTG or S113L mutation in exon 3 of the CPT II gene (a: patient; b: mother of the patient; c: normal control).

During hospitalization, the diagnosis of CPT II deficiency was suspected and the patient was referred for genetic analyses. Sequencing analysis of exons 1, 3, and 4 of the CPT II gene revealed that the patient was homozygote for Ser 113 Leu mutation (Figure 2). Diagnosis of adult myopathic form of carnitine palmitoyltransferase II deficiency was made based on the genetic findings and signs and symptoms as well as the age of onset.

## 3. DNA Sequencing

DNA was extracted from a peripheral blood sample using QiAmp DNA Blood Mini Kit (Qiagen). Exons 1, 3, and 4 of the CPT II gene were amplified by PCR using the following oligonucleotide primer pairs: CPT2 Ex 1 F: 5′-ACTCCAGAACTCCCCACTTG-3′/CPT2 Ex 1 R: 5′- CGGGTTCACTAGAGGAGTCA-3′ (299 bp PCR fragment); CPT2 Ex 3 F: 5′-CCTCGCCATGAACCTAAAAA-3′/CPT2 Ex 3 R: 5′-TTCATTATGGAGGGCTCTGG-3′ (231 bp PCR fragment); CPT2 Ex 4 F: 5′-CCCATTAAGGACCTTGTCCA-3′, CPT2 Ex 4 R: 5′-GCCTCAGAGCACCTCTTTGT-3′ (564 bp PCR fragment). Direct sequencing was performed at 3500 Series AB Genetic Analyzer (Applied Biosystems, Foster City, CA, USA).

## 4. Discussion

Carnitine palmitoyltransferase (CPT) CPT I deficiency is a very rare condition and is recognized in three different forms, of which the last two have not been identified in humans yet (might be incompatible with life): liver type CPT I deficiency; muscle-type and brain-type CPT I deficiency [1–3].

The CPT II deficiency presents in three different forms:

- lethal neonatal form,
- severe infantile hepatocardiomuscular form, and
- adult-myopathic form [1–6].

The lethal neonatal form is characterized by episodes of liver failure, hypoketotic hypoglycemia, cardiomyopathy, cardiac arrhythmias, seizures and coma after fasting or infection, and facial and structural abnormalities [4]. This presentation is fatal; the CPT II activity is barely detectable in any of the tissues, leading to death in the neonatal period [2, 7].

The severe infantile hepatocardiomuscular form is characterized by liver failure, cardiomyopathy, seizures, hypoketotic hypoglycemia, peripheral myopathy, and attacks of abdominal pain. The onset is more often before one-year of age and usually ends with a sudden death secondary to paroxysmal heart beat disorders [2, 4, 8].

The adult-myopathic form is the most prevalent type of the disease with about 300 cases reported. The majority of the patients are males (~80%) and it is inherited in an autosomal recessive manner. The first description was made in 1973 by the brothers Di Mauro. The first symptoms most often occur between 6 and 20 years of age but the age of onset may be over 50 years and as early as 8 months of life [9, 10]. The symptomatology usually consists of recurrent attacks of rhabdomyolysis presenting as myalgias, muscle stiffness and weakness, and myoglobinuria. The frequency of the symptoms is highly variable and they resolve within hours to days with clinically symptom-free periods between attacks. The rhabdomyolysis may occasionally be complicated by three kinds of life-threatening events, that is, acute renal failure due to myoglobinuria, respiratory insufficiency secondary to respiratory muscles involvement, and paroxysmal heart arrhythmias [11, 12]. The symptoms are usually provoked by prolonged exercise, fasting, high fat intake, exposure to cold, infections accompanied by fever, and general anesthesia and drugs such as ibuprofen, diazepam, and valproic acid. In general, the patients have normal life expectancy with complications such as acute kidney failure in massive rhabdomyolysis, usually sparing other organs. In our patient we observed cardiac complications.

Our patient presented with all the typical signs and symptoms, including a respiratory failure combined with heart failure, probably worsened by volume overload during the uric period of the disease. The findings of the echocardiography examinations done three years earlier and at present time identify dilated cardiomyopathy with a reduced ejection fraction. The strain analysis using speckle tracking showed average GLPS decrease of 13.8%. These findings could be prescribed to the nature of the disease and consequence of lipid storage in the myocardium even though unspecific for this form of disease. Could myocardial biopsy help us with our hypothesis? Comparisons with the results obtained three years ago showed a relatively stable course of heart failure (no significant progression of the left ventricle dimension or reduction of the ejection fraction).

Diagnosing CPT II deficiency can be done by acylcarnitine analysis using tandem mass spectrometry (peak at C16 is indicative of the condition). Measurement of the CPT II activity can be performed as well as many laboratory findings, such as low carnitine levels, increased serum creatinine kinase, and transaminase, which can be associated with the disease. For a definitive diagnosis, sequencing of the CPT II gene for a mutation analysis is recommended. The CPT II gene has been assigned to chromosome 1p32. It contains five exons and more than 25 mutations have been described [3]. The S113L is the most common mutation in the myopathic form. There is evidence that the S113L mutation leads to a thermolabile CPT II protein whose activity is reduced when the body temperature is high, as with exercise, fever, or heat stress [13].

Definitive diagnosis in our patient was established with the CPT II gene sequencing for mutation analysis, and the Ser 113 Leu mutation in exon 3 of the CPT II gene was identified (Figure 2). Genetic analyses were performed on the patient's family (mother and two siblings). We found that his mother, brother, and sister are carriers for the mutation, but none of them described any myalgia or symptoms suggestive of rhabdomyolysis.

The treatment of this condition consists of hygiene-dietary changes that would prevent attacks and symptomatic treatment of myoglobinuria and possible renal complications. Patients are advised to reduce the intake of long-chain dietary fat and increase the consumption of meals rich in carbohydrates. They should have more frequent meals and avoid fasting. Other things that should be avoided include prolonged exercise as well as some drugs (ibuprofen, diazepam, valproic acid, and general anesthesia). During intercurrent infections infusion of glucose can be administered. Oral carnitine supplementation can be considered as an adequate therapy. The medium-chain fatty acid triheptanoin may be effective in the adult-onset CPT II deficiency.

At present, nine months after this episode, our patient is clinically stable and no episodes of rhabdomyolysis have been registered. He is on heart failure treatment medications, and he is following the advice given to him concerning his life style modification. He does not work as a lorry driver anymore.
